# Correction: A data-driven approach to establishing cell motility patterns as predictors of macrophage subtypes and their relation to cell morphology

**DOI:** 10.1371/journal.pone.0346789

**Published:** 2026-04-07

**Authors:** Manasa Kesapragada, Yao-Hui Sun, Kan Zhu, Cynthia Recendez, Daniel Fregoso, Hsin-Ya Yang, Marco Rolandi, Rivkah Isseroff, Min Zhao, Marcella Gomez

The Data Availability statement for this article is incorrect. The correct statement is: The complete dataset is available at https://doi.org/10.5061/dryad.m0cfxpp8v with a metadata file accompanying the dataset. The source code required to reproduce the findings of this study has been uploaded to the following GitHub repository: https://github.com/Gomez-Lab/CellAnalysis.
