# Supplementary figures and images for: A data-driven approach to establishing cell motility patterns as predictors of macrophage subtypes and their relation to cell morphology
Source: PLoS One. 2024 Dec 31;19(12):e0315023. doi: 10.1371/journal.pone.0315023 (PMC11687909; doi:10.1371/journal.pone.0315023)

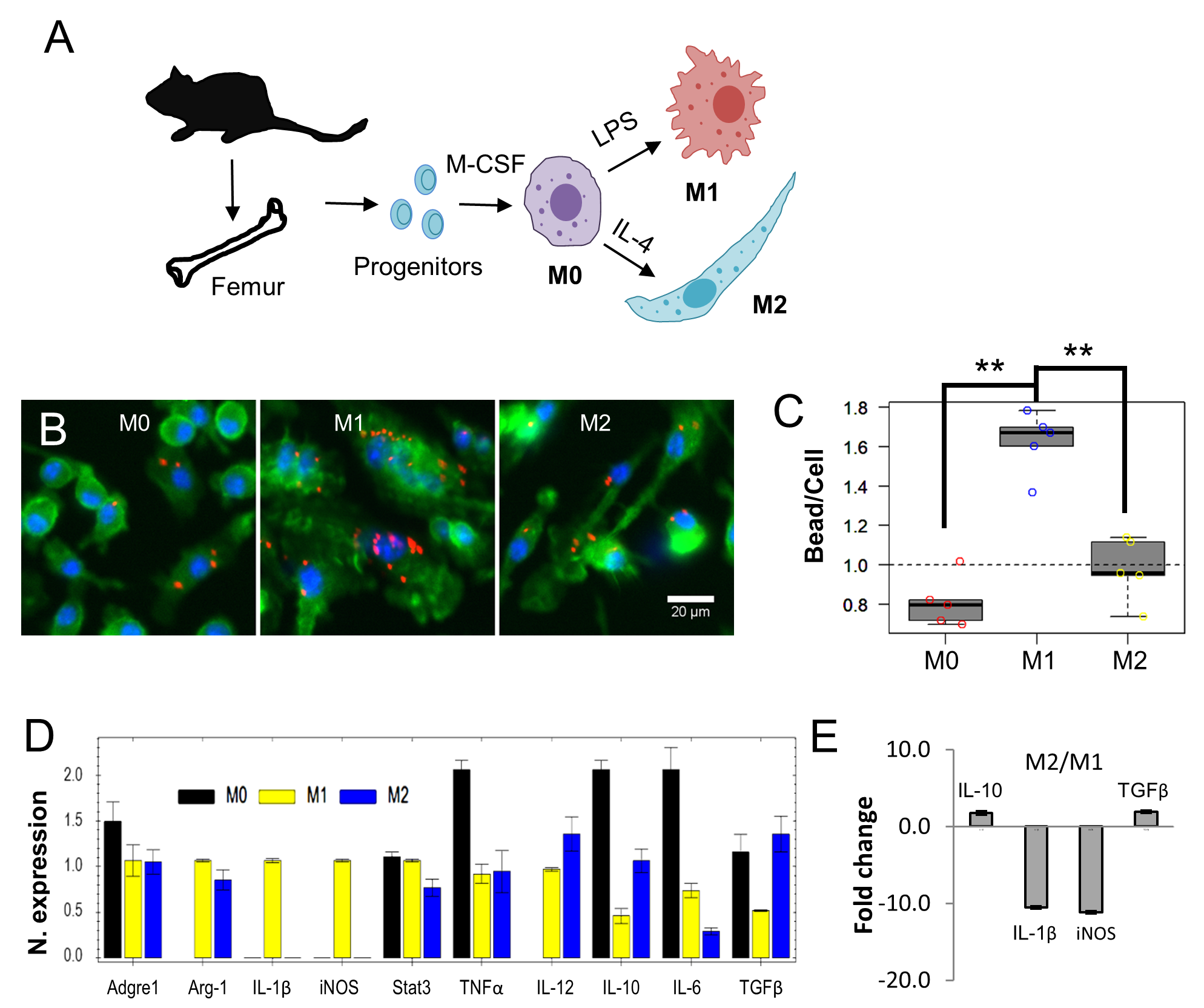

Supplement: S1 Fig — (A) Scheme for the generation of mouse bone marrow derived macrophages (BMDMs, M0) and subsequent polarization of M1 (100 ng/ml LPS) and M2 (20 ng/ml mIL-4) macrophages. (B) Fluorescent images show M0, M1 and M2 macrophages containing latex beads (red). Actins were stained with FITC-phalloidin (green). Nuclei were counterstained with Hoechst (blue). Note the significantly increased phagocytosis capacity of M1 macrophages. Bar, 20 μm. (C) Quantification of phagocytosis. Data was calculated as bead per cell from 5 randomly chosen fields. ** p < 0.01 by one-way ANOVA with post-hoc Tukey HSD Test. (D) The target gene expression profiles of naïve and differentially polarized macrophages. Fold expression is calculated relative to the internal control of GAPDH mRNA expression. (E) Differential M1 (IL-1β and iNOS) or M2 marker (IL-10 and TGFβ) gene expressions induced by LPS or mIL-4, respectively. Fold change is calculated as M2/M1 ratio of mRNA expression. (TIF) [file pone.0315023.s001.tif]

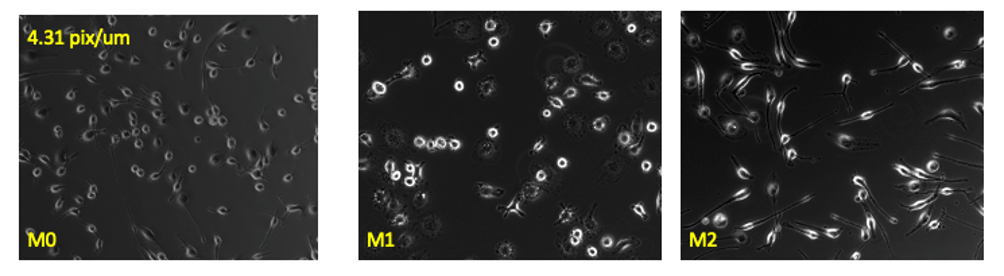

Supplement: S2 Fig — (TIF) [file pone.0315023.s002.tif]

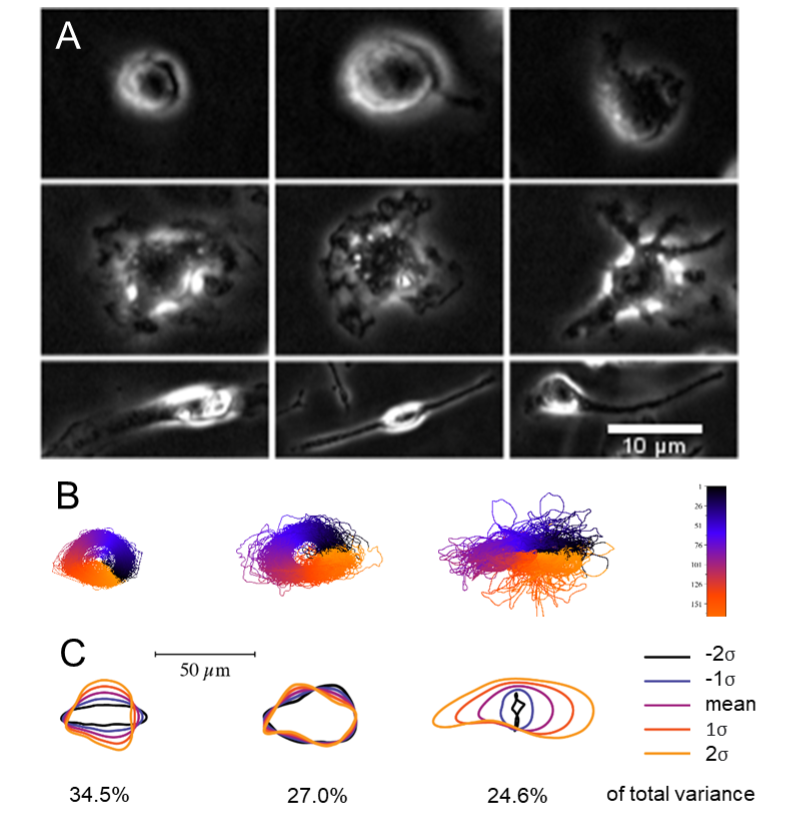

Supplement: S3 Fig — (A) Phase-contrast images of differentially polarized macrophages illustrate the phenotypic shape variation in the combined population. Bar, 10 μm. (B) The alignments of 2329 live macrophages by their outlines that are equally divided into 200 points. (C) Top three principal modes of macrophage shape variation as determined by principal components analysis. These modes—circular (mode 1), “with protrusions” shape (mode 2, one example is shown), and elongated (mode 3)—are highly reproducible; subsequent modes seem to be mixtures or noise. For each mode, the mean cell shape is shown alongside reconstructions of shapes one and two standard deviations away from the mean in each direction along the given mode. The variation accounted for by each mode is indicated. Bar, 50 μm. (TIF) [file pone.0315023.s003.tif]

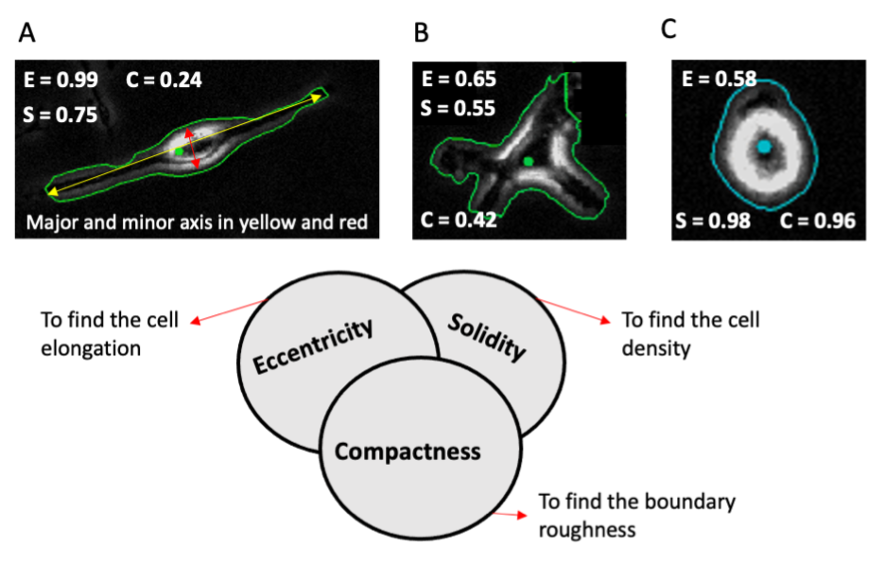

Supplement: S4 Fig — A) Elongated/Bipolar cell with compactness = 0.24, eccentricity = 0.99, solidity = 0.75—highest eccentricity, with low compactness and higher solidity, (B) Multipolar cell with compactness = 0.42, eccentricity = 0.65, solidity = 0.55—average eccentricity, with low compactness and solidity, (C) Circular cell with compactness = 0.96, eccentricity = 0.58, solidity = 0.98—average eccentricity, with highest compactness and solidity. (TIF) [file pone.0315023.s004.tif]

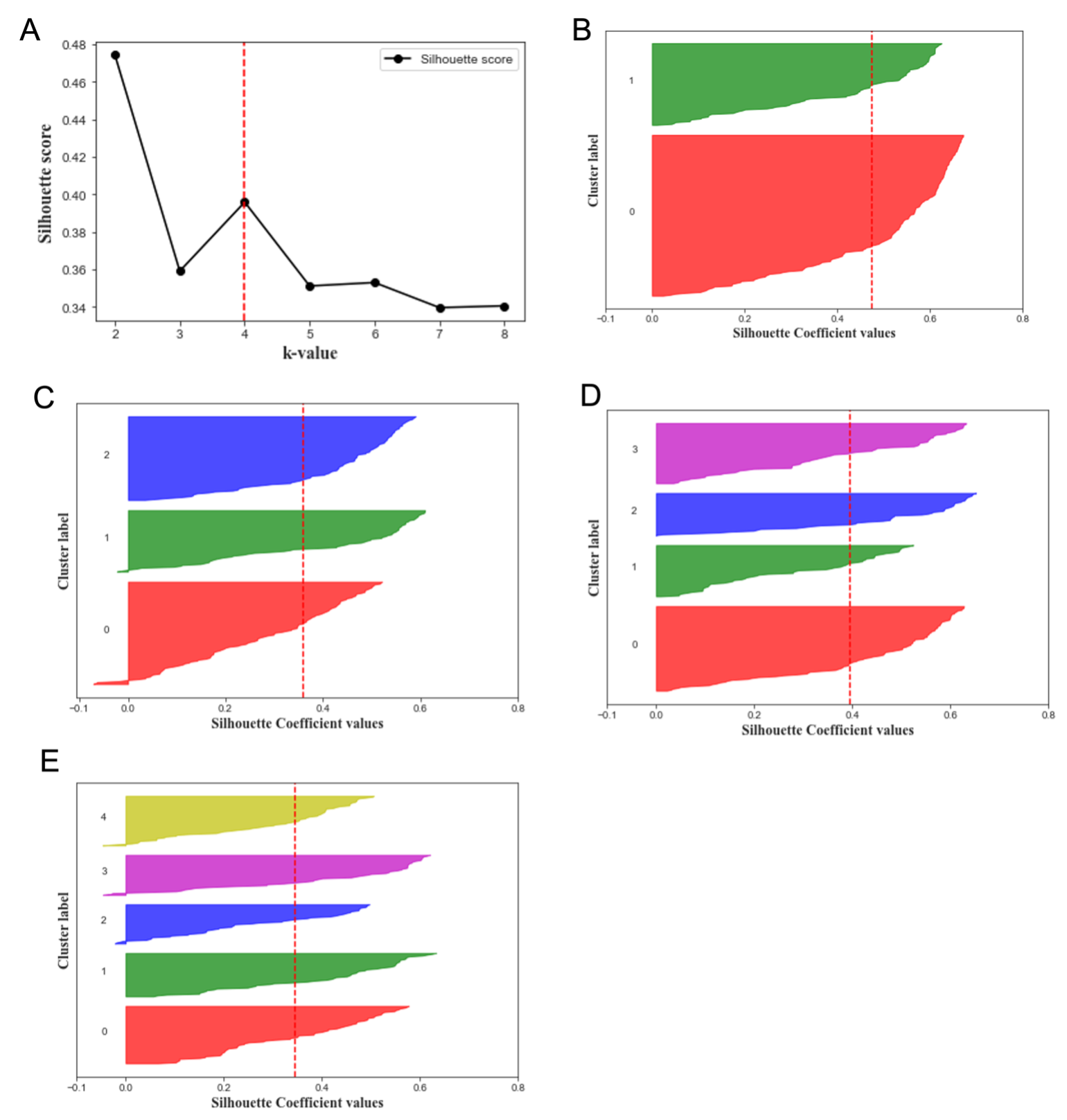

Supplement: S5 Fig — A) Silhouette score plot with highest values for k = 2 and k = 4, (B) Silhouette plot for k = 2 with irregular cluster thickness showing one cluster is bigger in size than the other—suboptimal k-value, (C) Silhouette plot for k = 3 with negative values in two of the clusters indicating cells are assigned to wrong clusters and below average score- suboptimal k-value, (D) Silhouette plot for k = 4 with Silhouette score higher than k = 3 and k = 5, without any negative values and somewhat uniform cluster thickness—optimal k-value, (E) Silhouette plot for k = 5 with negative values in three of the clusters indicating cells are assigned to wrong clusters and below average score—suboptimal k-value. (TIF) [file pone.0315023.s005.tif]

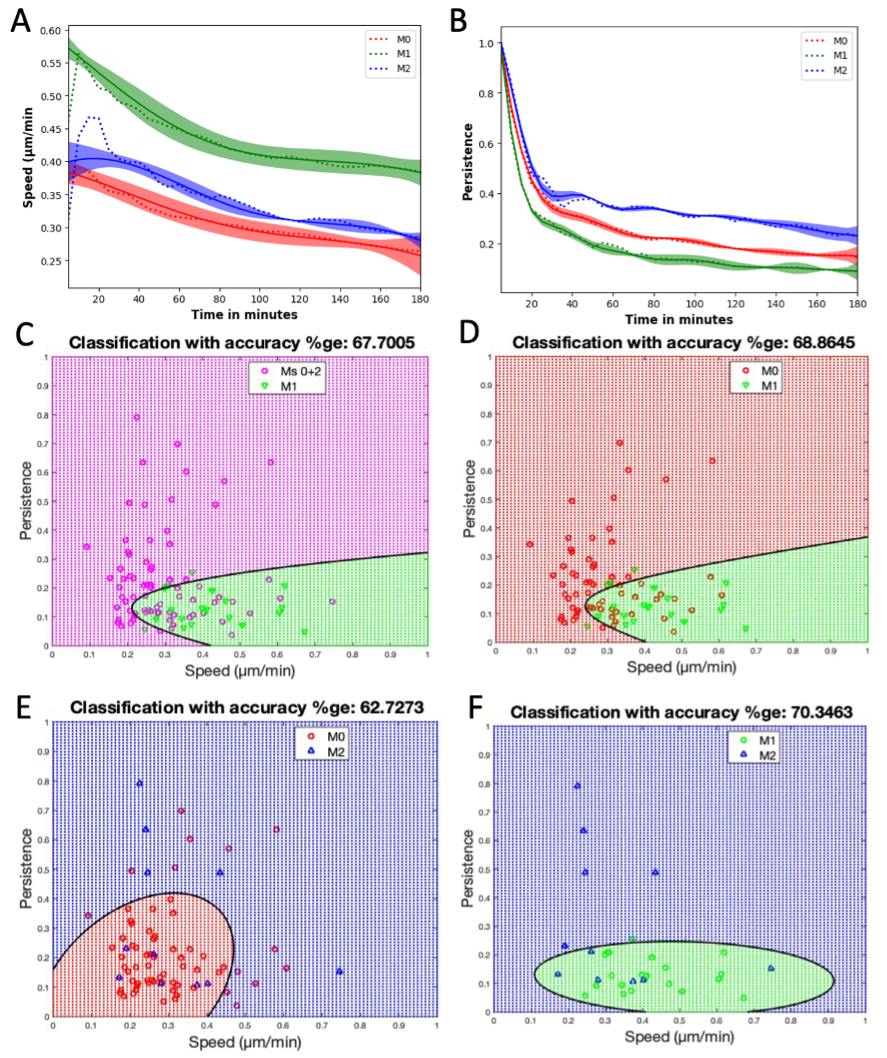

Supplement: S6 Fig — (A) Speed, (B) Persistence, where dotted lines are the mean values of the corresponding macrophage images, solid lines are the Gaussian Process Regressor model (GPR) predictions, and the color bands are their 95% confidence intervals. (C) Plot showing the quadratic classification of the cells using Speed vs Persistence between M1 image and combined (M0+M2) images with a classification accuracy of around 68%. (D) Plot showing the quadratic classification of the cells using Speed vs Persistence between M0 image and M1 image with a classification accuracy of around 69%. (E) Plot showing the quadratic classification of the cells using Speed vs Persistence between M0 image and M2 image with a classification accuracy of around 63%. (F) Plot showing quadratic the classification of the cells using Speed vs Persistence between M1 image and M2 image with a classification accuracy of around 70%. The black line in C-F is the decision boundary between the classes. (TIF) [file pone.0315023.s006.tif]

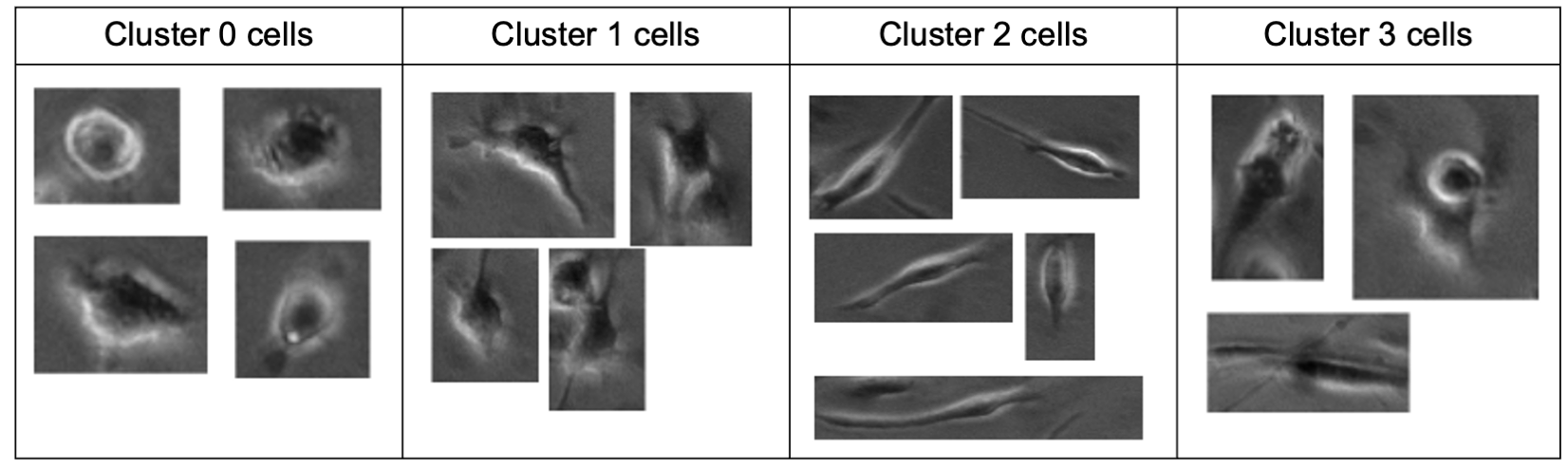

Supplement: S7 Fig — (TIF) [file pone.0315023.s007.tif]
