# Supplementary material for: A data-driven approach to establishing cell motility patterns as predictors of macrophage subtypes and their relation to cell morphology
Source: PLoS One. 2024 Dec 31;19(12):e0315023. doi: 10.1371/journal.pone.0315023 (PMC11687909; doi:10.1371/journal.pone.0315023)
Supplement: S1 File — (PDF) [file pone.0315023.s008.pdf]

Supporting information

Generation and characterization of primary murine macrophages

Macrophage phagocytosis assay

Phagocytosis is a major function of macrophages to capture and kill pathogens, as well as to remove apoptotic cells or cell debris (45). We confirm the phagocytic capacity of the naive BMDMs and LPS or IL-4 primed M1 or M2 macrophages by challenging them with fluorescently labelled latex beads (**Fig. S1*B***). While polarized M1 and M2 macrophages were both found to have enhanced phagocytic capacity, it is worth noting that the M1 macrophage that were primed by pathogenic bacterial LPS had a significantly higher number of internalized latex beads (1.624 ± 0.070, bead/cell) when compared with M0 macrophages or M2 macrophages primed by IL-4 (0.811 ± 0.057 and 0.979 ± 0.072, respectively) (**Fig. S1*C***), as has also been reported by other groups (46).

BMDMs were seeded on 12 mm cover slips that were pre-coated with FNC Coating Mix (Athena, Cat number: 0407) for 4 hours at RT in 24-well plate (2×10^5 cells per well) and cultured in RPMI-1640 (Invitrogen) with 10% Fetal Bovine Serum (Invitrogen) and 1× Antibiotic-Antimycotic solution (Invitrogen) for overnight. Macrophages were activated by following procedures as described above. Fluorescently labeled microspheres (ThermoFisher, Cat number: F8821) were used to challenge macrophages at a multiplicity of infection of 20 (20 beads per cell). After 60 min of incubation, cells were gently washed 3× with PBS to remove excessive and un-adherent beads, and further incubated in culture media in the absence (for M0) or presence of LPS (for M1) or IL-4 (for M2) for additional 16 h. To quantify phagocytosis, macrophages were fixed with 4% paraformaldehyde and permeabilized with 0.1% Tryton-X100 in PBS. F-actin were labelled by FITC-conjugated Phalloidin (Sigma, Cat number: P5282). Nuclei were labeled by Hoechst 33342 (Sigma, Cat number: 94403). Cells were photographed using a Leica TCS SP8 confocal microscope (Leica microsystem). Images were processed using ImageJ. Quantification and comparison of fluorescent intensity were done in images taken in the

same batch with the same optical setup and parameters. Beads and nuclei were identified by thresholding and counted using particle analysis function.

Quantitative real-time PCR

To further characterize the phenotype of polarized macrophages, we performed quantitative PCR to determine the relative mRNA expression of a panel of selected target genes and transcription factors.

Total RNAs were isolated from naïve M0, LPS-simulated M1 or IL-4-stimulated M2 macrophages using the Trizol- (Life Technologies Cat # 15596026) chloroform method, followed by RNeasy Mini Kit (Qiagen). cDNA was synthesized by QuantiTect Reverse Transcription Kit (Qiagen). qRT-PCRs were performed on the ViiA 7 Real-Time PCR System (Applied Biosystems) using PowerUp SYBR Green Master Mix (ThermoFisher) according to the manufacturer's instructions. Primer sequences are listed in **S2 Table**. Relative expression values were normalized by internal housekeeping genes (GAPDHs). Fold changes were calculated as ratios of M2 *versus* M1 macrophages.

S1 Fig. (D) shows typical profiles and variations of macrophage polarization-associated genes of unpolarized and differentially polarized macrophages by LPS or IL-4 for 48 h. Specifically, the transcription levels of IL-1β and inducible nitric oxide synthase (iNOS), two well-established markers of proinflammatory macrophages, are increased more than 10 folds in LPS stimulated M1 macrophages compared to that of IL-4 stimulated M2 macrophages. In contrast, the transcriptions of IL-10 and transforming growth factor beta (TGFβ), two characterized pro-healing macrophage markers, are significantly higher in M2 macrophages, compared to that of M1 macrophages (**S1 Fig. (E)**). Thus, we have generated and polarized primary macrophages with characterized phenotypes that are applicable and appropriate for the proposed downstream studies and analysis.

Shape modeling with principal component analysis

Phase contrast images of M0, M1 and M2 macrophages were converted into the binary images using custom written Matlab code (Script available upon request). Briefly, we used Matlab edge detection and a basic morphology function to outline the cells in the phase contrast image. We

use the Otsu method (47) to erase the halo artifacts. If shape was still unsatisfactory, we then used the Lasso tool in Photoshop (Adobe) to manually extract the cell shape. For principal component analysis, polygons were extracted from the binary images and mutually aligned using Celltool (28), an open software source. Principle modes of shape variation were determined by principal component analysis of the population of polygonal cell outlines and scaled in terms of the standard deviation of the population for each mode of variation, which was detailed previously (48). To identify the existing principal shape modes, we consider each cell in every frame of the time-lapse datasets as individual data points (n = 2329 cells). We obtain the shape information for M0 (n = 965 cells), M1 (n = 698 cells) and M2 (n = 666 cells) (**S3 Fig. (B)**). These segmentations are not used in downstream analysis.

Preliminary Motility Analysis

We use the Gaussian process regressor model from the Scikit-learn python library (32) to plot the speed and persistence of the cells. We found that the speed of the cells in the M1 image is highest, followed by the speed of the cells in M2 and the lowest speed observed for the cells in M0 (**S6 Fig. (A)**). In contrast, the persistence of the cells in M2 is highest, followed by the persistence of the cells in M0 and the lowest persistence observed for the cells in M1 (**S6 Fig. (B)**). Based on this motility parameter analysis, we conclude that:

- M0 cells move slower and stay close to their initial time frame position.
- M1 cells move faster but still stay close to their initial time frame position.
- M2 cells move slower but reach farther away from their initial time frame position.

To better visualize and quantify the motility of the cells in M0, M1 and M2 images, we perform quadratic discriminant analysis on the median of the cells speed and persistence along its time series and plot them. This analysis provides a binary classification based on two input features (**S6 Fig.**, showing the results for various pairs of classification labels). Classification accuracy percentages show that the cells in M1 image can be distinguished combined or independently from M0 and M2 cells using the speed and persistence motility parameters (**S4 Figs. (C), (D)**, **(F)**). Notably, the classification accuracy percentage between M0 and M2 cells is comparatively lower around 63%, suggesting that it is comparatively difficult to classify M0 cells from M2 cells (**S6 Fig. (E)**).
