# Supplementary material for: A data-driven approach to establishing cell motility patterns as predictors of macrophage subtypes and their relation to cell morphology
Source: PLoS One. 2024 Dec 31;19(12):e0315023. doi: 10.1371/journal.pone.0315023 (PMC11687909; doi:10.1371/journal.pone.0315023)
Supplement: S1 Table — (PDF) [file pone.0315023.s009.pdf]

Table S1. Macrophage subtype classification with accuracy percentages for different analysis techniques used in the study

**Input parameters**	**Method**	**M0**	**M1**	**M2**
Morphology	K-means Clustering	38%	52%	46%
Morphology	SVM	69%	71%	71%
Motility	SVM	75%	75%	41%
**Morphology + Motility**	SVM	**81%**	**79%**	**79%**
