# Supplementary material for: A data-driven approach to establishing cell motility patterns as predictors of macrophage subtypes and their relation to cell morphology
Source: PLoS One. 2024 Dec 31;19(12):e0315023. doi: 10.1371/journal.pone.0315023 (PMC11687909; doi:10.1371/journal.pone.0315023)
Supplement: S2 Table — (PDF) [file pone.0315023.s010.pdf]

Table S2: Target genes and primers used in q-PCR.

Target gene	Forward primers (5'-3')	Reverse primers (5'-3')	Macrophage Subtypes
Arg-1	CGTTGTATGATGCACAGCCG	CCCCACCCAGTGATCTTGAC	M1
CD206	GTTCACCTGGAGTGATGGTTCTC	AGGACATGCCAGGGTCACCTTT	M2
CD31	ACAGAGCCAGCAGTATGA	AATGACAACCACCGCAAT	Angiogenesis
CXCL10	GATGGATGGACAGCAGAG	GGAAGATGGTGGTTAAGTTC	M1
Adgre1	TCTGGGGAGCTTACGATGGA	GAATCCCGCAATGATGGCAC	Macrophage
Fizz-1	CCTGCTGGGATGACTGCTACT	AGATCCACAGGCAAAGCCAC	M2
GAPDH	TCTCCTGCGACTTCAACA	TGTAGCCGTATTCATTGTCA	Housekeeping
IL-10	CAGAGCCACATGCTCCTAGA	TGTCCAGCTGGTCCTTTGTT	M2
IL-12p40	TGGTTTGCCATCGTTTTGCTG	ACAGGTGAGGTTCACTGTTTCT	M1
IL-1β	GTGCAAGTGTCTGAAGCAGC	CAAAGGTTTGGAAGCAGCCC	M1
IL-6	GGAGTCACAGAAGGAGTGGC	CGCACTAGGTTTGCCGAGTA	M2
iNOS	GTTCTCAGGCCAACAATACAAGA	GTGGACGGGTCGATGTCAC	M1
MCP-1	AGCCAACTCTCACTGAAGCC	GGACCCATTCCTTCTTGGGG	M1
MMP-9	CTGGACAGCCAGACACTAAAG	CTCGCGGCAAGTCTTCAGAG	M2 MMP
PPAR-γ	TCCTGTAAAAGCCCGGAGTAT	GCTCTGGTAGGGGCAGTGA	M2 Metabolic
Stat3	CTTGTCTACCTCTACCCCGACAT	GATCCATGTCAAACGTGAGCG	M1 TF
Stat6	TGAGGTGGGGACCAGCCGG	GTGACCAGGACACACAGCGG	M2 TF
TGF-β1	TGGAGCAACATGTGGAACTC	CAGCAGCCGGTTACCAAG	M2
TLR4	CAGAACAATAGAAGAGGAAGAC	GGCACTAACCACATAGAGAA	M1
TNF-α	GACGTGGAACTGGCAGAAGA	ACTGATGAGAGGGAGGCCAT	M1
Ym-1	CTCACTTCCACAGGAGCAGG	AGCTGCTCCATGGTCCTTC	M2
β-actin	GGCTGTATTCCCCTCCATCG	CCAGTTGGTAACAATGCCATGT	Housekeeping
